# An Application of Machine Learning to Etiological Diagnosis of Secondary Hypertension: Retrospective Study Using Electronic Medical Records

**DOI:** 10.2196/19739

**Published:** 2021-01-25

**Authors:** Xiaolin Diao, Yanni Huo, Zhanzheng Yan, Haibin Wang, Jing Yuan, Yuxin Wang, Jun Cai, Wei Zhao

**Affiliations:** 1 Department of Information Center Fuwai Hospital Chinese Academy of Medical Sciences and Peking Union Medical College Beijing China; 2 Department of Information Center, Fuwai Hospital National Center for Cardiovascular Diseases Chinese Academy of Medical Sciences and Peking Union Medical College Beijing China; 3 Hypertension Center, State Key Laboratory of Cardiovascular Disease, Fuwai Hospital National Center for Cardiovascular Diseases Chinese Academy of Medical Sciences and Peking Union Medical College Beijing China

**Keywords:** secondary hypertension, etiological diagnosis, machine learning, prediction model

## Abstract

**Background:**

Secondary hypertension is a kind of hypertension with a definite etiology and may be cured. Patients with suspected secondary hypertension can benefit from timely detection and treatment and, conversely, will have a higher risk of morbidity and mortality than those with primary hypertension.

**Objective:**

The aim of this study was to develop and validate machine learning (ML) prediction models of common etiologies in patients with suspected secondary hypertension.

**Methods:**

The analyzed data set was retrospectively extracted from electronic medical records of patients discharged from Fuwai Hospital between January 1, 2016, and June 30, 2019. A total of 7532 unique patients were included and divided into 2 data sets by time: 6302 patients in 2016-2018 as the training data set for model building and 1230 patients in 2019 as the validation data set for further evaluation. Extreme Gradient Boosting (XGBoost) was adopted to develop 5 models to predict 4 etiologies of secondary hypertension and occurrence of any of them (named as composite outcome), including renovascular hypertension (RVH), primary aldosteronism (PA), thyroid dysfunction, and aortic stenosis. Both univariate logistic analysis and Gini Impurity were used for feature selection. Grid search and 10-fold cross-validation were used to select the optimal hyperparameters for each model.

**Results:**

Validation of the composite outcome prediction model showed good performance with an area under the receiver-operating characteristic curve (AUC) of 0.924 in the validation data set, while the 4 prediction models of RVH, PA, thyroid dysfunction, and aortic stenosis achieved AUC of 0.938, 0.965, 0.959, and 0.946, respectively, in the validation data set. A total of 79 clinical indicators were identified in all and finally used in our prediction models. The result of subgroup analysis on the composite outcome prediction model demonstrated high discrimination with AUCs all higher than 0.890 among all age groups of adults.

**Conclusions:**

The ML prediction models in this study showed good performance in detecting 4 etiologies of patients with suspected secondary hypertension; thus, they may potentially facilitate clinical diagnosis decision making of secondary hypertension in an intelligent way.

## Introduction

Hypertension is a common chronic disease worldwide, with 5%-10% of these patients being secondary hypertensive [1-5]. Patients with secondary hypertension who have high risks of morbidity and mortality if not diagnosed and treated timely are early onset cases, with higher blood pressure (BP) that is more difficult to be controlled than patients with primary hypertension [2-4,6]. Secondary hypertension identification is already known to benefit patients who have suggestive signs and symptoms, such as severe or resistant hypertension and an acute rise in BP from previously stable readings [1-3,5]. It is necessary to focus on accurate diagnosis to capture the secondary hypertension of patients in order to provide effective evidence for clinical therapy [2-4,7].

Artificial intelligence (AI) is seen as having the potential to provide more efficient medical services and has been applied in medical care, such as disease diagnosis, risk stratification, and health management [8-21]. AI technologies, especially machine learning (ML), have received attention in the diagnosis and treatment of hypertension. However, previous studies were focused on predicting future risks of hypertension and building clinical decision support systems to support early screening and treatment [22-31]. In addition, there are no relevant published studies on AI model–aided diagnosis of secondary hypertension for detecting etiologies of disease and providing effective treatment.

Accordingly, we used electronic medical record (EMR) data from Fuwai Hospital, a large, urban teaching hospital affiliated with Peking Union Medical College in Beijing, China, to develop ML diagnosis models of common etiologies of secondary hypertension and validate the feasibility and effectiveness of such models in assisting clinical diagnosis of secondary hypertension [32]. This study, based on representative and nationwide in-patient data, is ideally positioned to generate information to construct diagnosis-aided models for secondary hypertension during hospitalization.

## Methods

### Study Population

**Figure 1:**
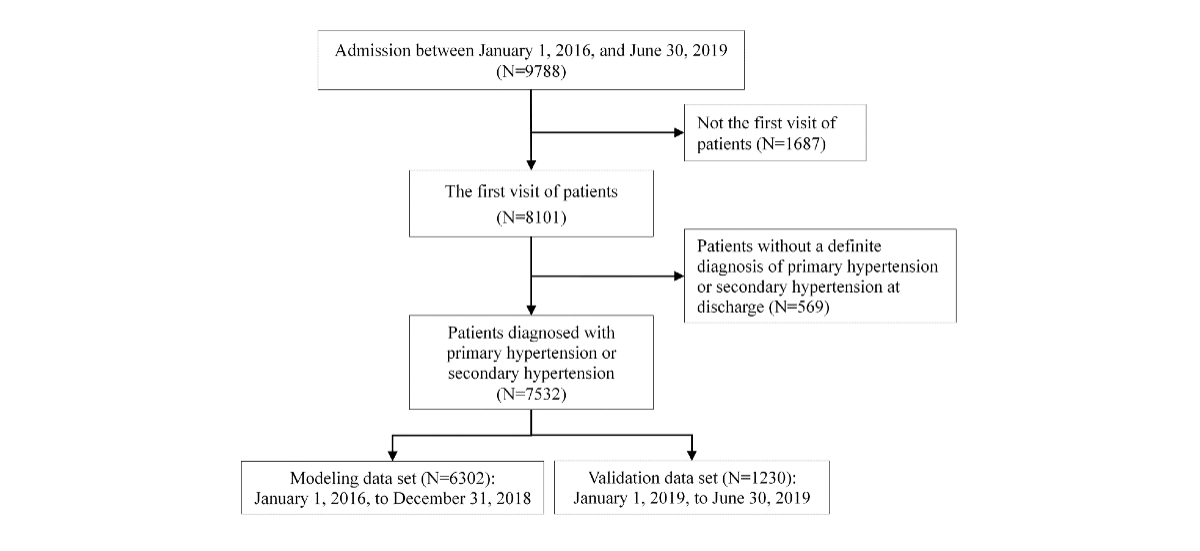
A workflow for patients inclusion and application.

Our study consecutively enrolled 9788 admissions from the Hypertension Center, Fuwai Hospital, from January 1, 2016, to June 30, 2019. The following data were collected: demographics, preadmission symptoms, comorbidities, medication history of antihypertension, operation history, physical examination indicators, prehospital and intrahospital BP, intrahospital first laboratory test results, and computed tomography (CT) reports. For multiple visits of patients, only the first visits were taken into consideration, so we excluded 1687 re-admission records. A total of 569 patients without a definite diagnosis of primary hypertension or secondary hypertension at discharge were also excluded. The final analyzed data set included 7532 unique patients and was divided into 2 mutually exclusive data sets by time: 6302 patients in 2016-2018 as the modeling data set for feature selection and model building, and 1230 patients in 2019 as the validation data set for subsequent evaluation and external verification (Figure 1). This study was approved by the Ethics Committee at Fuwai Hospital with the requirement for informed consent waived. Data used in this study were anonymous, and no identifiable personal data of the patients were used.

### Outcome Definitions

Etiologies of secondary hypertension in this study were defined by the International Classification of Diseases, 10th Revision, Clinical Modification (ICD-10-CM) diagnosis codes. Prediction models were developed for the following 5 outcomes chosen by the incidence rate: (1) renovascular hypertension (RVH), assigned the ICD-10-CM diagnosis code I15.001; (2) primary aldosteronism (PA), assigned the ICD-10-CM diagnosis code I15.201; (3) thyroid dysfunction, assigned the ICD-10-CM diagnosis codes E03.901 and E05.901; (4) aortic stenosis, assigned the ICD-10-CM diagnosis codes Q25.101, Q25.301, I77.102, I77.112, and I77.122; (5) composite outcome, defined as occurrence of any of (1)-(4).

### Data Processing

We computed the maximum, minimum, and range among prehospital and intrahospital BP cases, respectively. The structured CT information was extracted from CT text reports using regular expressions and was standardized based on uniform medical terminology in cardiovascular medicine used in Fuwai Hospital. The capping method was used to deal with outliers in order to avoid the model performance being affected by potential input errors, and to retain most of the information. When there were missing values, we created an additional binary variable that assigned a value of 1 if missing and 0 otherwise. All continuous variables were converted to categorical variables by the smbinning package of R 3.4.4 software (R Foundation), which was a supervised binning method based on the conditional inference tree. All categorical variables were one-hot coded [33].

### Feature Selection

Two kinds of feature selection methods were introduced successively in our study. First, we used univariate logistic analysis to eliminate features that were unlikely to predict the outcomes with a *P*-value threshold of .01. Then, we randomly split modeling data set into training data set and test data set by 8:2, and conducted Gini Impurity to rank the contribution of features and only keep the top 20% of features as the final features for each outcome based on the training data set.

### Model Building

**Figure 2:**
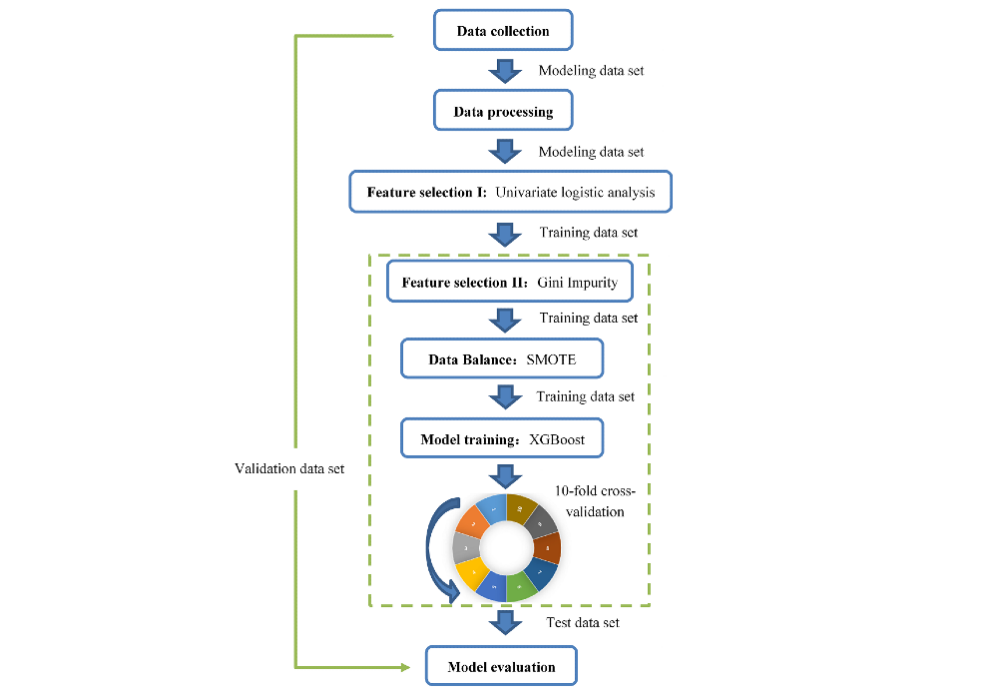
Procedure flow of modeling. SMOTE: Synthetic Minority Oversampling Technique; XGBoost: extreme Gradient Boosting.

Five ML models of 4 etiologies of secondary hypertension and 1 composite outcome were trained using the training data set. Before training, the synthetic minority oversampling technique was adopted to deal with the unbalanced issue of the training data set [34]. XGBoost (Extreme Gradient Boosting), an ensemble tree-based model, has been shown to be more likely to achieve better model performance and to be more interpretable than other ML models, such as logistic regression or support vector machine [35-39]. Therefore, we choose the XGBoost algorithm to develop the prediction model for each outcome. In order to avoid overfitting, we used grid search and 10-fold cross-validation to select the optimal hyperparameters (Figure 2).

For all outcomes, we compared the receiver operating characteristic curve and the area under the curve (AUC), accuracy, sensitivity, specificity, and precision to measure model performance in the test data set of the modeling data set and the validation data set. Furthermore, the accuracy of the composite outcome model on different age subgroups (≤18, 19-44, 45-59, and ≥60) was evaluated. All analyses were performed using R software version 3.4.4 (R Foundation for Statistical Computing).

## Results

### Baseline Characteristics

Of the 7532 patients included in this study, 64.82% (4882/7532) were male, with a mean age of 47.70 (SD 14.77), a mean maximum systolic pressure of 173.00 (SD 29.50) mmHg, and a mean maximum diastolic pressure of 124.87 (SD 32.56) mmHg. Among them, 72.48% (5459/7532) were diagnosed with hypertension in the past, and 6.70% (505/7532), 5.31% (400/7532), 1.85% (139/7532), and 0.94% (71/7532) were diagnosed with RVH, PA, thyroid dysfunction, and aortic stenosis at discharge, respectively. As much as 13.95% (1051/7532) of patients were diagnosed with any of the 4 etiologies at discharge (ie, with composite outcome). Most characteristics were similarly distributed between the 2 data sets (Table 1).

**Table 1:** Baseline characteristics.

Characteristic		Modeling data set (N=6302)		Validation data set (N=1230)		All data set (N=7532)	
Male, n (%)		4089 (64.88)		793 (64.47)		4882 (64.82)	
Age (years), mean (SD)		47.74 (14.80)		47.48 (14.61)		47.70 (14.77)	
BMI (kg/m^2^), mean (SD)		26.47 (3.69)		26.62 (3.75)		26.49 (3.70)	
Maximum SP^a^ (mmHg), mean (SD)		172.57 (29.96)		175.20 (26.96)		173.00 (29.50)	
Minimum SP (mmHg), mean (SD)		110.46 (28.95)		107.99 (29.72)		110.06 (29.09)	
Maximum DP^b^ (mmHg), mean (SD)		124.15 (32.85)		128.53 (30.77)		124.87 (32.56)	
Minimum DP (mmHg), mean (SD)		79.45 (12.62)		79.14 (12.55)		79.40 (12.61)	
**Comorbidities**							
	Hypertension, n (%)		4938 (78.36)		521 (42.36)		5459 (72.48)
	Hyperlipemia, n (%)		2846 (45.16)		486 (39.51)		3332 (44.24)
	Cerebrovascular disease, n (%)		1007 (15.98)		158 (12.85)		1165 (15.47)
	Thyroid disease, n (%)		462 (7.33)		72 (5.85)		534 (7.09)
	Hypokalemia, n (%)		106 (1.68)		24 (1.95)		130 (1.73)
**Medication history of** **antihypertension**							
	Nifedipine, n (%)		2056 (32.62)		400 (32.52)		2456 (32.61)
	Amlodipine, n (%)		1776 (28.18)		340 (27.64)		2116 (28.09)
	Verapamil hydrochloride, n (%)		1621 (25.72)		605 (49.19)		2226 (29.55)
	Metoprolol, n (%)		1545 (24.52)		244 (19.84)		1789 (23.75)
	Enalapril maleate, n (%)		346 (5.49)		50 (4.07)		396 (5.26)
**Discharge diagnosis**							
	RVH^c^, n (%)		409 (6.49)		96 (7.80)		505 (6.70)
	PA^d^, n (%)		323 (5.13)		77 (6.26)		400 (5.31)
	Thyroid dysfunction, n (%)		119 (1.89)		20 (1.63)		139 (1.85)
	Aortic stenosis, n (%)		59 (0.94)		12 (0.98)		71 (0.94)
	Composite outcome, n (%)		858 (13.61)		193 (15.69)		1051 (13.95)

^a^SP: systolic pressure.

^b^DP: diastolic pressure.

^c^RVH: renovascular hypertension.

^d^PA: primary aldosteronism.

### Model Performance

**Figure 3:**
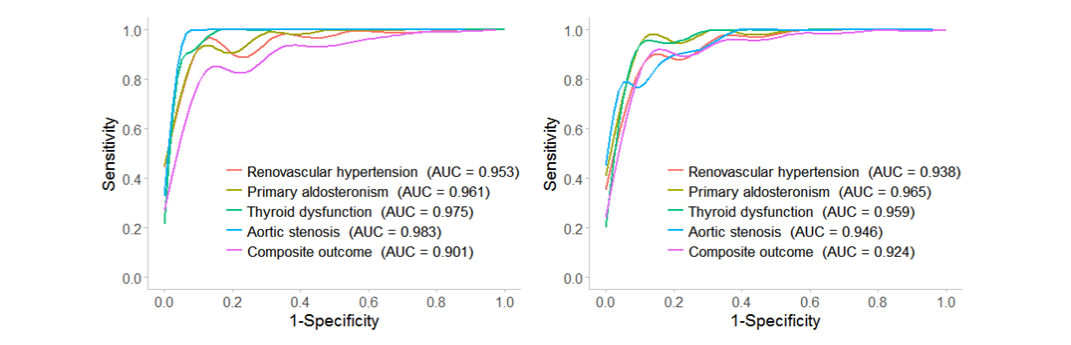
ROC curves for prediction models in both data sets. (A) ROC curves for prediction models in the test data set of the modeling data set. (B) ROC curves for prediction models in the validation data set. AUC: area under ROC; ROC: receiver-operating characteristic curve.

The 4 prediction models of secondary hypertension etiologies reached AUCs of 0.953-0.983 with sensitivities of 83.6%-92.9% and specificities of 89.9%-95.9% in the test data set of the modeling data set, whereas they achieved AUCs of 0.938-0.965 with sensitivities of 75.0%-90.0% and specificities of 89.4%-97.3% in the validation data set. Among them, the prediction model of PA achieved the best model performance with AUC of 0.965, sensitivity of 84.4%, specificity of 93.0%, and precision of 44.5% in the validation data set. The prediction model of composite outcome showed good performance in the test data set of the modeling data set with an AUC, sensitivity, specificity, and precision of 0.901, 82.1%, 84.6%, and 45.8%, respectively, as well as in the validation data set with values of 0.924, 85.5%, 86.2%, and 53.6%, respectively (Figure 3 and Table 2).

**Table 2:** Model performance.

Outcomes		AUC^a^	Accuracy, %	Sensitivity, %	Specificity, %	Precision, %
**RVH^b^**						
	Test data set	0.953	90.0	87.1	90.2	41.5
	Validation data set	0.938	88.9	83.3	89.4	40.0
**PA^c^**						
	Test data set	0.961	95.3	83.6	95.9	47.9
	Validation data set	0.965	92.4	84.4	93.0	44.5
**Thyroid dysfunction**						
	Test data set	0.975	90.0	92.9	89.9	17.3
	Validation data set	0.959	92.5	90.0	92.6	16.7
**Aortic stenosis**						
	Test data set	0.983	95.5	90.0	95.5	13.8
	Validation data set	0.946	97.1	75.0	97.3	21.4
**Composite outcome**						
	Test data set	0.901	84.2	82.1	84.6	45.8
	Validation data set	0.924	86.1	85.5	86.2	53.6

^a^AUC: area under the receiver-operating characteristic curve.

^b^RVH: renovascular hypertension.

^c^PA: primary aldosteronism.

### Impactful Features

A total of 362 clinical indicators were considered initially and a total of 79 indicators were finally included in our 5 prediction models, 46 of which were included in the prediction model of composite outcome, and 33, 21, 14, and 14 were included in the prediction model of RVH, PA, thyroid dysfunction, and aortic stenosis, respectively. The remaining indicators included 2 demographic indicators, 3 preadmission symptoms, 5 BP indicators, 4 comorbidities, 5 antihypertension medications, 2 operation indicators, 3 physical examination indicators, 46 intrahospital first laboratory tests, and 9 indicators from CT reports (Multimedia Appendix 1). Each of the 4 prediction models of secondary hypertension etiologies had their own typical indicators of high contribution while only a few indicators were included in at least two prediction models. The indicators used in the composite outcome prediction model were mainly derived from the most important indicators of 4 etiology prediction models (Table 3).

**Table 3:** Top 10 clinical indicators for prediction models.

Clinical indicators		Contribution^a^, %
**RVH^b^**		
	Renal artery stenosis indicated by CT^c^	67.9
	Abnormalities of renal artery indicated by CT	3.4
	Albumin-to-creatinine ratio^d^	2.7
	NT-proBNP^e^	2.7
	Cerebrovascular disease^f^	2.2
	Abnormalities of adrenal glands indicated by CT	2.1
	Maximum systolic pressure	1.9
	Creatine kinase	1.7
	The level of renal artery stenosis indicated by CT	1.3
	Glutamyl transpeptidase	1.2
**PA^g^**		
	Upright ARR^h^	49.7
	Serum potassium	17.9
	Supine ARR	5.6
	Supine plasma aldosterone	3.9
	Upright plasma aldosterone	2.8
	Glycated hemoglobin	2.7
	Nifedipine	2.4
	Albumin-to-creatinine ratio	2.3
	24-hour urinary aldosterone	2.3
	Serum sodium	2.1
**Thyroid dysfunction**		
	Thyroid disease	60.1
	Thyrotropin	28.5
	Prealbumin	1.7
	Free thyroxine	1.4
	Range of systolic pressure	1.2
	Metoprolol	1.2
	Palpitation	1.2
	Surgery	1.0
	Dizzy	1.0
	Thyroid microsomal antibody	0.9
**Aortic stenosis**		
	Carotid bruits	22.2
	Age	22.1
	Vascular bruits	20.2
	BMI	12.9
	Aortic wall thickening or stenosis indicated by CT	5.6
	Upright plasma renin	5.2
	Smoking status	3.9
	Glomerular filtration rate	3.7
	Supine plasma aldosterone	1.6
	Range of systolic pressure	0.9
**Composite outcome**		
	Renal artery stenosis indicated by CT	26.9
	Upright ARR	16.5
	Thyroid disease	10.0
	Serum potassium	6.0
	Albumin-to-creatinine ratio	4.4
	Supine ARR	3.4
	Supine plasma aldosterone	2.5
	Nifedipine	2.5
	Hemoglobin concentration	1.9
	Maximum systolic pressure	1.9

^a^The contribution represents the proportion of the information gain of each indicator in the total information gain of all indicators. The total contribution of all indicators included in each prediction model is 1. The higher the contribution, the more important the indicator in the model.

^b^RVH: renovascular hypertension.

^c^CT: computed tomography.

^d^All the laboratory test indicators were the first intrahospital laboratory test data of patients.

^e^NT-proBNP: N-terminal probrain natriuretic peptide.

^f^All the symptoms and medical and treatment history were reported by patients themselves upon admission.

^g^PA: primary aldosteronism.

^h^ARR: aldosterone-to-renin ratio.

### Subgroup Analysis

The validation of the composite outcome prediction model in different age groups showed good discrimination with AUCs greater than 0.8 in all groups and sensitivities greater than 80% in all groups of adults (Table 4). It should be noted that sensitivity in minors only achieved 66.7%, which is mainly because there were not enough samples of minors included in this study.

**Table 4:** Model performance of the composite outcome prediction model in different age groups.

Metrics	Minors (≤18 years) (N=29)	Youth (19-44 years) (N=502)	Middle aged (45-59 years) (N=406)	Elderly (≥60 years) (N=293)
AUC^a^	0.833	0.943	0.912	0.895
Accuracy, %	89.7	92.0	82.3	80.9
Sensitivity, %	66.7	89.1	87.3	82.2
Specificity, %	92.3	92.3	81.2	80.5
Precision, %	50.0	53.9	49.6	58.3

^a^AUC: area under the receiver-operating characteristic curve.

## Discussion

### Principal Results

Based on the EMRs from Fuwai Hospital, we developed 5 prediction models with good performance for 4 etiologies of secondary hypertension using XGBoost. Validation of the composite outcome prediction model achieved an AUC of 0.924, while the 4 prediction models of the secondary hypertension etiologies achieved AUCs of 0.938-0.965 in the validation data set. The observed model performance suggested that it was feasible to derive effective ML prediction models of secondary hypertension, which may play important roles in predicting etiologies of patients with suspected secondary hypertension.

### Comparison With Prior Work

With the accumulation, integration, and standardization of medical information, as well as the constant improvement of computing power, the potential uses for AI in medicine are growing [40]. AI-assisted diagnosis is a very important medical application field and its application in hypertension has gained attention [22-27]. Some studies of AI technologies in the prediction and diagnosis of hypertension or primary hypertension have been published; for instance, a real-time risk prediction model of future 1-year incident essential hypertension using XGBoost has been deployed in Maine, providing inspiration for hypertension and related disease intervention [26]. Detection of secondary hypertension is of great significance in the clinical diagnosis and treatment of hypertension. Chinese guidelines for the prevention and treatment of hypertension state that all patients with hypertension need undergo the assessment of secondary hypertension [4]. Nonetheless, no studies regarding AI-assisted diagnosis in secondary hypertension have been published yet. Our study filled this gap and will potentially be useful in enhancing the detection of etiologies of secondary hypertension.

All patients included in this study needed to consider the possibility of secondary hypertension according to the admission criteria of patients with hypertension in Fuwai Hospital, which ensured that the prediction models were applicable to detection of extensive etiologies of secondary hypertension [7]. Compared to ML prediction models in previous similar studies, it can be seen that the prediction models derived from this study showed good performance [41-46]. The models in our study achieved AUCs of 0.924-0.965 in the validation data set. Furthermore, validation of the composite outcome prediction model on different age groups has been performed, which demonstrated high discrimination in all age groups of adults.

Most of the features identified in this study were consistent with those of the previous studies [1,2,4,5,47-51]. It has been reported that the main imaging methods for the diagnosis of renal artery stenosis were CT, magnetic resonance imaging, and ultrasound [5]. Both albumin-to-creatinine ratio and NT-proBNP were important indicators of renal function [47,51], which are also of great significance for RVH prediction in our model. Aldosterone-to-renin ratio was a screening tool for PA [2,48]. Our model indicated that serum potassium played an important role in the PA prediction model [4,49]. Besides thyroid disease, thyrotropin and free thyroxine were the core clinical indicators for identification of thyroid dysfunction [1]. One of the main clinical manifestations of aortic stenosis is carotid bruits [4]. In addition, there was a certain correlation between age and aortic stenosis which has been demonstrated in previous studies [1,50].

### Application of the Prediction Models

Application of ML methods to etiological diagnosis of secondary hypertension can be useful in clinical practice. As the use of EMRs is becoming increasingly common in hospitals, it is convenient to obtain an individual’s integrated clinical data [26]. ML algorithms can comprehensively analyze all the obtained information of patients, and will be more targeted and flexible than traditional guidelines. AI technology should be implemented cautiously, as to be partners, or even mentors of clinicians, there is still a long way to go, but it can serve as a virtual assistant and enable clinicians to promote quality and improve efficiency. The ML prediction models derived from our study hold promise for developing a diagnostic tool for detection of secondary hypertension and integration into EMR systems to offer real-time clinical support. Model reasoning will be invoked automatically and the most probable etiology of secondary hypertension will be recommended for clinical reference. Moreover, it will be of great significance to apply the diagnostic models, based on big data of authoritative medical institutions, to community medical institutions. The practice results manifested that the models developed in this study have the potential to realize this vision after further optimization and prospective verification.

### Limitations

There are several limitations of this study. It is worth noting that not all common secondary hypertension etiologies were covered in this study; however, we are making efforts to accumulate more data and expand the samples and indicators to accomplish and add more etiological prediction models. Direct text analysis for extracting CT features is language specific; therefore, the models must be adapted and revised before using them in a different language setting. Lastly, more external validations are in need and will be performed with more different data sets.

### Conclusions

Based on the EMRs from Fuwai Hospital, 5 ML prediction models with good performance and applicable to etiologies detection of secondary hypertension in all age groups of adults were developed, which demonstrated that ML approaches were feasible and effective in the diagnosis of secondary hypertension. Such prediction models have the potential to help clinical decision making which may augment and extend effectiveness of the clinicians and help to develop more intelligent, more efficient, and more convenient hypertension diagnosis modes. However, these innovative and clinically relevant prediction models still require further validation and more clinical tests before being implemented into clinical practice.

## Appendix

Multimedia Appendix 1 The final 79 clinical indicators included in 5 prediction models and their contributions in each model. ARR: aldosterone-to-renin ratio; CT: computed tomography; NT-proBNP: N-terminal pro-brain natriuretic peptide; PA: primary aldosteronism; RVH: renovascular hypertension.

## Abbreviations

AI: artificial intelligence
AUC: area under the receiver-operating characteristic curve
BP: blood pressure
CT: computed tomography
EMR: electronic medical record
ICD-10-CM: International Classification of Diseases, 10th Revision, Clinical Modification
ML: machine learning
NT-proBNP: N-terminal pro-brain natriuretic peptide
PA: primary aldosteronism
RVH: renovascular hypertension
XGBoost: extreme Gradient Boosting
